# A CRISPR-Cas-based recombinase polymerase amplification assay for ultra-sensitive detection of active *Trypanosoma brucei evansi* infections

**DOI:** 10.3389/fmolb.2025.1512970

**Published:** 2025-02-14

**Authors:** Andrés Álvarez-Rodríguez, Zeng Li, Bo-Kyung Jin, Benoit Stijlemans, Peter Geldhof, Stefan Magez

**Affiliations:** ^1^ Lab of Cellular and Molecular Immunology, Brussels Center for Immunology (BCIM), Vrije Universiteit Brussel, Brussels, Belgium; ^2^ Department of Biochemistry and Microbiology (WE10), Ghent University, Ghent, Belgium; ^3^ Myeloid Cell Immunology Lab, VIB Center for Inflammation Research, Brussels, Belgium; ^4^ Laboratory for Parasitology and Parasitic Diseases, Department of Translational Physiology, Infectiology and Public Health (DI04), Ghent University, Ghent, Belgium

**Keywords:** Trypanosoma brucei evansi, Surra, CRISPR-Cas, RPA, diagnosis

## Abstract

**Introduction:**

Control of *Trypanosoma brucei evansi* (*T. b. evansi*) infections remains a significant challenge in managing Surra, a widespread veterinary disease affecting both wild and domestic animals. In the absence of an effective vaccine, accurate diagnosis followed by treatment is crucial for successful disease management. However, existing diagnostic methods often fail to detect active infections, particularly in field conditions. Recent advancements in CRISPR-Cas technology, combined with state-of-the-art isothermal amplification assays, offer a promising solution. This approach has led us to the development of a *Tev*RPA-CRISPR assay, a highly sensitive and specific *T. b. evansi* diagnostic tool suitable for both laboratory and field settings.

**Methods:**

First, the *Tev*CRISPR-Cas12b cleavage assay was developed and optimized, and its analytical sensitivity was evaluated. Next, this technology was integrated with the *Tev*RPA to create the *Tev*RPA-CRISPR test, with the reaction conditions being optimized and its analytical sensitivity and specificity assessed. Finally, the test’s accuracy in detecting both active and cured *T. b. evansi* infections was evaluated.

**Results:**

The optimized *Tev*CRISPR-Cas12b cleavage assay demonstrated the ability to detect *T. b. evansi* target DNA at picomolar concentrations. Integrating *Tev*CRISPR-Cas12b with RPA in Two-Pot and One-Pot *Tev*RPA-CRISPR tests achieved up to a 100-fold increase in analytical sensitivity over RPA alone, detecting attomolar concentrations of *T. b. evansi* target DNA, while maintaining analytical specificity for *T. b. evansi*. Both assays exhibited performance comparable to the gold standard *Tev*PCR in experimental mouse infections, validating their effectiveness for detecting active infections and assessing treatment efficacy.

**Discussion:**

The *Tev*RPA-CRISPR tests prove highly effective for diagnosing active infections and assessing treatment efficacy, while being adaptable for both laboratory and field use. Thus, the *Tev*RPA-CRISPR assays emerge as a promising addition to current diagnostic tools, offering efficient and reliable detection of active *T. b. evansi* infections.

## Introduction

*Trypanosoma brucei evansi* (*T. b. evansi*) is a hemoflagellate parasite causing Surra, the most widespread trypanosomal disease, primarily affecting domestic and wild animals including camels, cattle, buffaloes, horses, pigs, or deer (Kim et al., 2023; Li et al., 2020). Unlike other subspecies of *Trypanosoma brucei* (*T. brucei*) such as *T. b. rhodesiense* and *T. b. gambiense* (the causative agents of the human disease Sleeping Sickness), *T. b. evansi* has evolved to rely on mechanical transmission via biting flies (e.g., *Tabanus*, *Glossina, Stomoxys*, *Haematopoda*, *Chrypsos* or *Lyperosia*) or mammals (e.g., *Desmodus rotundus*) (Brun et al., 1998; Austen and Barbosa, 2021; Pays et al., 2023). This adaptation has allowed *T. b. evansi* to spread beyond the geographical constraints of tsetse-transmitted trypanosomes, widening its geographical distribution beyond Africa (Lun and Desser, 1995). Hence, it is prevalent in Asia, Africa, and South America, and has occasionally even been reported in Europe (Aregawi et al., 2019; Gutierrez et al., 2010). While *T. b. evansi* has traditionally been considered non-infective to humans, reports exist of atypical Human Trypanosomiasis (aHT) cases in Vietnam, India, and Sri Lanka, thereby highlighting the potential zoonotic risk associated with this parasite (Powar et al., 2006; Joshi et al., 2005; Van Vinh Chau et al., 2016). This situation is currently exacerbated by climate change-driven redistribution of vectors, as well as the encroachment of grazing areas into wildlife reservoirs, resulting in heightened human-parasite interactions (Mojahed et al., 2022; Kasozi et al., 2021).

In the absence of a vaccine against *T. b. evansi* trypanosomosis, current recommended control measures depend on accurate diagnosis, followed by individualized treatment of the infected animals (Radwanska et al., 2008). Available diagnostic tests for *T. b. evansi* differentiate between *T. b. evansi* type A, characterized by the presence of the Rode *Trypanozoon* antigenic type 1.2 Variant Surface Glycoprotein (RoTat1.2 VSG) gene (Ngaira et al., 2004), or *T. b. evansi* type B, characterized by the absence of that gene (Birhanu et al., 2016). While *T. b. evansi* type B trypanosomosis is restricted to certain regions in Africa (Birhanu et al., 2016; Ngaira et al., 2005; Behour and Abd El Fattah, 2023; Njiru et al., 2006; Salim et al., 2011; Boushaki et al., 2019), *T. b. evansi* type A is spread worldwide (Aregawi et al., 2019; Gutierrez et al., 2010). Diagnosis of *T. b. evansi* infections involves direct visualization of the parasite, detection of parasite-induced host antibodies (Abs), or detection of parasite nucleic acids (Tehseen et al., 2015). While microscopy-based techniques can effectively detect *T. b. evansi* parasites in infected samples, their use is limited to the acute stage of infection, failing to identify both latent and chronic stages when parasitemia is low (Behour et al., 2019). Additionally, these techniques require specialized equipment and trained personnel to ensure reliability, which limits their feasibility for point-of-care (POC) field testing. Therefore, the current standard protocol for assessing possible *T. b. evansi* infections recommends the use of antibody-based tests such as the Card Agglutination Test (CATT/*T. b. evansi*), the Latex Agglutination Test (LATEX/*T. b. evansi*), and the Enzyme-Linked Immunosorbent Assay (ELISA/*T. b. evansi*) (Pathak et al., 1997; Lejon et al., 2005; Reyna-Bello et al., 1998; WOAH, 2021). These tests are indeed useful for field testing but cannot discriminate between previous exposure to *T. b. evansi* or current infections. The presence of parasite-induced host Abs may indicate an active or past infection, but it can also result from repeated exposure to the parasite without successful infection or from polyclonal B cell activation due to other infectious agents (Harris and Gause, 2011; Fikru et al., 2015). Moreover, these tests often exhibit low specificity due to cross-reactions with Abs against closely related parasites, resulting in low specificity and low positive predictive values (PPV) (Fikru et al., 2015; Alvarez-Rodriguez et al., 2022). Consequently, the World Organization for Animal Health recommends the verification of the previous test results by specific Polymerase Chain Reaction (PCR) amplification in a controlled laboratory setting (WOAH, 2021). Recommended specific PCR primers include RoTat1.2 for *T. b. evansi* type A, and EVAB for *T. b. evansi* type B (WOAH, 2021). PCR detection of parasite DNA is effective at all stages of infection and allows the assessment of subsequent treatment effectiveness and the cure of infected animals (Li et al., 2020; Davila et al., 2003; Masiga et al., 1992). Nevertheless, this technique is limited to the use in lab settings mainly due to the need for specialized temperature control devices and well-trained technicians. As a solution, isothermal amplification methods for *T. b. evansi* have been recently developed as a good alternative to PCR. These include *T. b. evansi* type A and B Loop-Mediated Isothermal Amplification (LAMP) assays, which can be run at around 65°C for 1 h (Tong et al., 2018; Njiru et al., 2010), or *T. b. evansi* type A Recombinase Polymerase Amplification (RPA), which can be run at around 39°C for 30 min (Li et al., 2020). These assays, while optimal for both lab and field settings, do eventually suffer from non-specific amplifications resulting in lower sensitivity and specificity as compared to the gold standard PCR (Zou et al., 2020).

**FIGURE 1:**
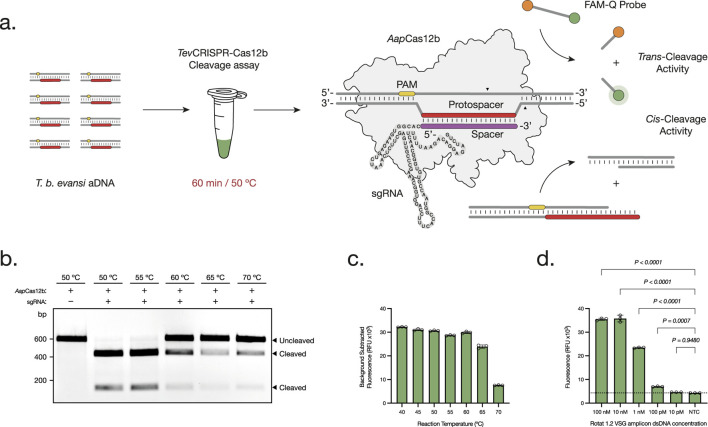
Development and Optimization of the *Tev*CRISPR-Cas12b cleavage assays. **(A)** The *Tev*CRISPR-Cas12b cleavage assay is performed in a 60 min reaction at 50°C. First, the ribonucleoprotein CRISPR-*Aap*Cas12b complex is formed between the *Aap*Cas12b protein and the single-guide RNA (sgRNA). Then, the Cas protein scans through the double-stranded DNA amplicon for the presence of a specific 5′-TTN-3′ Protospacer Adjacent Motif (PAM). Upon PAM recognition, the spacer region of the sgRNA hybridizes with the complementary protospacer sequence adjacent to the PAM site. As a result, the RuvC endonuclease domain of *Aap*Cas12b is activated, leading to the *cis*-cleavage of both DNA strands. This leads to the non-specific *trans*-cleavage of the FAM-Q probes, resulting in a measurable fluorescence signal of the released FAM reporters (Teng et al., 2019). **(B, C)** Temperature range analysis of both *Tev*CRISPR-Cas12b *cis*- **(B)** and *trans*-cleavage assays **(C)**. The *cis*-cleavage results were visualized on a 1% agarose gel pre-stained with ethidium bromide (t = 60 min). The *trans*-cleavage results were plotted as the background subtracted fluorescence of mean ± standard deviation (SD) of 3 technical replicates (t = 120 min). **(D)** Analytical sensitivity assessment of the *Tev*CRISPR-Cas12b cleavage assay. Background subtracted fluorescence of 3 technical replicates is plotted as mean ± SD. A Cut-off (NTC average +3 times the SD) is indicated by the dashed line. All statistical analyses were conducted using a one-way ANOVA, followed by Dunnett’s multiple comparison test. Significant differences between groups are denoted with the corresponding p-values listed above.

In recent years, the emergence of CRISPR-Cas technology has marked a significant step forward towards the generation of improved versions of diagnostic tests (Sima et al., 2022). The CRISPR-Cas complex can be programmed by using a synthetic single guide RNA (sgRNA) fragment together with a Cas endonuclease to target and cleave a specific DNA/RNA sequence (Jinek et al., 2012) (Figure 1A). Many Cas proteins, upon their inherent *cis*-cleavage activity on the specific DNA/RNA target, display an additional non-specific *collateral* or *trans*-cleavage activity on surrounding single-stranded DNA/RNAs (Sereno et al., 2022; East-Seletsky et al., 2016; Gootenberg et al., 2017). This property has been effectively harnessed as a sensitive diagnostic approach to specifically identify nucleic acids present in a sample, by coupling current diagnostic tests together with the CRISPR-Cas complex and ssDNA/RNA probes containing fluorescent reporters (Chen et al., 2018; Li et al., 2018; Li et al., 2019). When combined with isothermal amplification methods, CRISPR-Cas-based diagnostic tests have demonstrated enhanced sensitivity and specificity, while preserving their usability for POC and Point of Need (PON) field testing (Zou et al., 2020; Lee et al., 2020; Cunningham et al., 2021; Ali et al., 2020).

In this study, we describe the development of the first CRISPR-Cas-based RPA Assay for the detection of active *T. b. evansi* infections (*Tev*RPA-CRISPR). We demonstrate the versatility and sensitivity of *Tev*CRISPR-Cas12b cleavage assays, showing how this technology outperforms the current *Tev*RPA assay when integrated into a combined *Tev*RPA-CRISPR test, and its accuracy in assessing both active and cured *T. b. evansi* infections.

## Materials and methods

### Nucleic acid preparations

Total genomic DNA (gDNA) from different *Trypanosoma* parasites (Table 1) was extracted and purified from infected mouse whole blood (at ∼10^8^ Trypanosomes mL^−1^) using the DNeasy Blood & Tissue Kit (Qiagen, Germany) following the manufacturer’s guidelines. DNA samples were eluted in DNase/RNase-free water and diluted to 1 ng μL^−1^ before storage at −20°C until further use. The concentration and quality of the purified total gDNA was assessed through gel electrophoresis and spectrophotometric analysis (performed on Nanodrop ND-1000, Thermo Scientific). To determine the analytical sensitivity of the Two-Pot and One-Pot tests with total gDNA, the extraction and purification of *T. b. evansi* RoTat 1.2 total gDNA was followed by a 1:10 serial dilution from 20 ng μL^−1^ up to 200 fg μL^−1^ with DNase/RNase-free water.

**TABLE 1:** Specifications of the *Trypanosoma* parasites employed in this study.

Strain	Host	Country
*T. b. gambiense* ANTAT 9.1	Human	Cameroon
*T. b. rhodesiense* STIB 850	Human	Uganda
*T. b. brucei* ANTAT 1.8	Bushbuck	Uganda
*T. b. equiperdum* BOTAT 1.1	Horse	Morocco
*T. b. evansi* ROTAT 1.2	Water Buffalo	Indonesia
*T. b. evansi* ANTAT 3.1	Capybara	South America
*T. b. evansi* KAZAKHSTAN	Camel	Kazakhstan
*T. b. evansi* COLOMBIA	Horse	Colombia
*T. b. evansi* VIETNAM	Water Buffalo	Vietnam
*T. b. evansi* MERZOUGA 93	Camel	Morocco
*T. b. evansi* MERZOUGA 56	Camel	Morocco
*T. b. evansi* ZAGORA I.17	Camel	Morocco
*T. b. evansi* ZAGORA II.28	Camel	Morocco
*T. b. evansi* ZAGORA III.25	Camel	Morocco
*T. b. evansi* CAN 86 K	Dog	Brazil
*T. b. evansi* STIB 816	Camel	China
*T. b. evansi* KETRI 2480	Camel	Kenya
*T. b. evansi* KETRI 2479	Camel	Kenya
*T. congolense* TRT 17	Cattle	Zambia
*T. vivax* ILRAD 700	Cattle	Nigeria
*T. cruzi* TULAHUEN	Arthropod	Chile

Total gDNA of *T. b. evansi* type A strains was used as a template to amplify by PCR a 615 bp fragment of the Rode *Trypanozoon* antigenic type 1.2 VSG (RoTat 1.2 VSG) gene (GenBank accession code: AF317914.1). The PCR amplification reaction was as follows: Ten μl of extracted total gDNA (at 1 ng μL^−1^) were mixed with 15 μL of a PCR-mastermix containing: 2 U GoTaq G2 DNA Polymerase (Promega, United Kingdom), 1x Colorless GoTaq Reaction Buffer (Promega, United Kingdom), 0.4 mM dNTPs (Thermo Fisher Scientific, United States), 0.8 μM *Tev*PCR-Fw primer (Integrated DNA Technologies, United States) and 0.8 μM *Tev*PCR-Rv primer (Integrated DNA Technologies, United States) (See primer sequence in Table 2). Amplifications were performed in a Biometra Trio-block thermocycler at the following cycling conditions: denaturation for 4 min at 94°C, followed by 35 amplification cycles of 1 min, denaturation at 94°C, 1 min primer-template annealing at 55°C, and 1 min polymerization at 72°C. A final elongation step was carried out for 5 min at 72°C. The resulting amplicon DNAs (aDNA) were purified with the GenElute PCR Clean-Up kit (Sigma-Aldrich) following the kit’s guidelines eluting in DNase/RNase-free water and diluted to 100 nM before storage at −20°C until further use. The concentration and quality of the purified aDNA were assessed through gel electrophoresis and spectrophotometric analysis (performed on a Nanodrop ND-1000, Thermo Scientific). To determine the analytical sensitivity of the CRISPR-Cas12b cleavage reactions and the Two-Pot and One-Pot tests with aDNA, *T. b. evansi* RoTat 1.2 aDNA was 1:10 serially diluted from 100 nM up to 1 aM with DNase/RNase-free water.

**TABLE 2:** Primers, probes and sgRNAs employed in this study.

Assay type	Primer name	Oligonucleotide (5′-3′)	Reference
PCR	*Tev*PCR-Fw	CACCGAAGCAAGCGCAGCAAGAG	This study
	*Tev*PCR-Rv	AGTTCCGGTACCTTCTCCATTTC	This study
*Tev*RPA	*Tev*RPA-Fw	CACCGAAGCAAGCGCAGCAAGAGGGTTAGCA	Li et al. (2020)
	*Tev*RPA-Rv	GTAGCTGTCTCCTGGGGCCGAGGTGTCATAG	Li et al. (2020)
*Tev*RPA-CRISPR Two-Pot and One-Pot	*Tev*RPA-Fw	CACCGAAGCAAGCGCAGCAAGAGGGTTAGCA	Li et al. (2020)
	*Tev*RPA-Rv	GTAGCTGTCTCCTGGGGCCGAGGTGTCATAG	Li et al. (2020)
	FAM-Q Probe	[6-FAM]TTTTT[BHQ-1]	This study
	RoTat1.2sgRNA	GUCUAGAGGACAGAAUUUUUCAACGGGUGUGCCAAUGGCCACUUUCCAG GUGGCAAAGCCCGUUGAGCUUCUCAAAUCUGAGAAGUGGCACUGUGGG CAAAGCCGACGGCA	This study
PCR	RoTat1.2 Fw	GCGGGGTGTTTAAAGCAATA	Class et al. (2004)
	RoTat1.2 Rv	ATTAGTGCTGCGTGTGTTCG	Class et al. (2004)

### *Tev*CRISPR-Cas12b *cis*-cleavage reactions

The recombinant *Alicyclobacillus acidiphilus* Cas12b (*Aap*Cas12b) protein, selected for the development of the *Tev*RPA-CRISPR tests, was purchased from SignalChem Diagnostics, Canada. The selected suitable sgRNA for this protein includes the *Alicyclobacillus acidoterrestris* Cas12b (*Aac*Cas12b) scaffold sgRNA (Supplementary Table S1), which given the absence of a published native *Aap*Cas12b sgRNA, results in a more robust and specific nuclease activity by the *Aap*Cas12b protein compared to other sgRNA scaffolds (Joung et al., 2020). All sgRNAs used in this study (Table 2; Supplementary Table S1) were synthesized by Integrated DNA Technologies, United States.

*Tev*CRISPR-Cas12b *cis*-cleavage assays were performed as follows: 250 nM *Aap*Cas12b, 500 nM RoTat1.2sgRNA and 30 nM *T. b. evansi* RoTat 1.2 aDNA were combined in 1x ThermoPol Reaction Buffer (New England Biolabs, United States) to a final volume of 15 μL. The reaction mix was transferred to a preset thermocycler and incubated for 1 h at different temperatures (50°C, 55°C, 60°C, 65°C, and 70°C). After incubation, the reaction was stopped by adding 2.75 μL of a stop solution (16.9 mM EDTA, 84.5 μg/mL RNAse A, and 67.6 mAU/mL proteinase K) and incubating the mix in a preset thermocycler for 10 min at 56°C. The reaction products were analyzed by electrophoresis on a 1% agarose gel pre-stained with ethidium bromide (EtBr) in TBE buffer (90 mM Tris, 90 mM borate, 2.5 mM EDTA). Electrophoresis was conducted at 100 V for 30 min.

### *Tev*CRISPR-Cas12b *trans*-cleavage reactions

*Tev*CRISPR-Cas12b *trans*-cleavage reactions were performed as follows: 62.5 nM Cas12b (or 62.5 nM, 120 nM, and 250 nM during optimization assays), 250 nM sgRNA (or 125 nM, 250 nM, 500 nM and 1,000 nM during optimization assays) (six different sgRNAs were assessed, see Table 2; Supplementary Table S1), 250 nM FAM-Q probe (Integrated DNA Technologies, United States) and 30 nM *T. b. evansi* RoTat 1.2 aDNA (or 5 uL of 100 nM, 10 nM, 1 nM, 100 pM and 10 pM initial concentration, for analytical specificity assessment) were combined in 1x ThermoPol Reaction Buffer (New England Biolabs, United States) to a final volume of 15 μL. The reaction mix was transferred to a preset thermocycler and incubated for 2 h at 50°C (or 50°C, 55°C, 60°C, 65°C and 70°C during optimization assays). The reactions were run using Hard-shell thin wall 96-well PCR Plates (Bio-Rad, United States) on the CFX Connect Real-Time PCR Detection System (Bio-Rad, United States). Fluorescence measurements were read every 30 s at λex: 493 nm, λem: 517 nm.

### Two-Pot *Tev*RPA-CRISPR test

Two-Pot *Tev*RPA-CRISPR tests include two consecutive reactions, being (i) the specific amplification of the target *T. b. evansi* DNA through RPA, and (ii) the specific detection of the amplicons through the CRISPR-Cas12b *cis*- and *trans*-cleavage activities.

Isothermal RPA amplification was conducted with the TwistAmp Basic kit (TwistDx, Cambridge, United Kingdom) with the protocol suggested by Li et al. (2020) with minor modifications: 10 μL of input aDNA (at 10 fM, 1 fM, 100 aM, 10 aM and 1 aM initial concentration) or total gDNA (at 20 ng μL^−1^, 2 ng μL^−1^, 200 pg μL^−1^, 20 pg μL^−1^, 2 pg μL^−1^ and 200 fg μL^−1^ initial concentration) were incubated with 480 nM of each *Tev*RPA primer, 1x rehydration buffer, 14 mM MgOAc and the lyophilized enzyme pellet of the TwistAmp Basic kit, in a final volume of 50 μL. The reaction mix was transferred to a preset thermocycler and incubated for 30 min at 39°C. The amplified products were first purified using the GenElute PCR Clean-Up kit (Sigma-Aldrich) and visualized by electrophoresis on a 2% agarose gel pre-stained with ethidium bromide (EtBr) in TBE buffer (90 mM Tris, 90 mM borate, 2.5 mM EDTA). Electrophoresis was conducted at 110 V for 40 min.

CRISPR-Cas12b specific detection was performed as follows: 2.5 μL of the previous reaction mix without purification was incubated with 62.5 nM *Aap*Cas12b, 250 nM RoTat1.2sgRNA, 250 nM FAM-Q probe (Integrated DNA Technologies, United States) and 1x ThermoPol Reaction Buffer (New England Biolabs, United States) in a final volume of 15 μL. The reaction mix was transferred to a preset thermocycler and incubated for 30–120 min at 50°C. The reactions were run using Hard-shell thin wall 96-well PCR Plates (Bio-Rad, United States) on the CFX Connect Real-Time PCR Detection System (Bio-Rad, United States). Fluorescence measurements were read every 30 s at λex: 493 nm, λem: 517 nm.

### One-Pot *Tev*RPA-CRISPR test

One-Pot *Tev*RPA-CRISPR tests combine two reactions in one single tube, being (i) the specific amplification of the target *T. b. evansi* DNA through RPA, and (ii) the specific detection of the amplicons through the CRISPR-Cas12b *cis*- and *trans*-cleavage activities.

For this assay, 5 μL of input aDNA (at 1 pM, 100 fM, 10 fM, 1 fM, 100 aM and 10 aM initial concentration) or total gDNA (at 20 ng μL^−1^, 2 ng μL^−1^, 200 pg μL^−1^, 20 pg μL^−1^ and 2 pg μL^−1^) was incubated with 480 nM of each *Tev*RPA primer, 1x rehydration buffer, 14 mM MgOAc, the lyophilized enzyme pellet of the TwistAmp Basic kit, 62.5 nM *Aap*Cas12b, 250 nM RoTat1.2sgRNA and 250 nM FAM-Q probe (Integrated DNA Technologies, United States) in a final volume of 15 μL. The reaction mix was transferred to a preset thermocycler and incubated for 60–120 at 39°C. The reactions were run using Hard-shell thin wall 96-well PCR Plates (Bio-Rad, United States) on the CFX Connect Real-Time PCR Detection System (Bio-Rad, United States). Fluorescence measurements were read every 30 s at λex: 493 nm, λem: 517 nm.

### Experimental mice infections

Eight-week-old male C57BL/6 mice (purchased from Janvier, France) were divided into two groups of six individuals. In each group, five mice were inoculated intraperitoneally with 2000 *T. b. evansi* STIB 816 parasites in 200 μL of HBSS buffer (140 mM NaCl, 5 mM KCl, 1 mM CaCl_2_, 0.4 mM MgSO_4_ 7H_2_O, 0.5 mM MgCl_2_ 6H_2_O, 0.3 mM Na_2_HPO_4_ 2H_2_O, 0.4 nM KH_2_PO_4_, 6 mM D-Glucose, 4 mM Sodium bicarbonate; Thermo Fisher Scientific, United States). Of note, bloodstream trypanosome parasites were stored at −80°C as blood aliquots containing 50% Alsever’s solution (Sigma–Aldrich) and 10% glycerol (final V/V). One mouse in each group was used as a negative control and was not infected. The mice were tail bled at different times post-infection. The mice in Group 1 were bled at days 1, 3, 5 and 7 post-infection. The animals in Group 2 were bled at days 0, 2, 4, 6, 8 and 10 post-infection. All individuals from Group 2 were treated with Berenil (40 mg per kg), administered intraperitoneally at day 5 post-infection. Blood samples were collected from the tail and mixed with heparinized saline (10-fold at 10 units/mL; Sigma-Aldrich, United States) to prevent coagulation. Then, 2.5 μL of the collected blood was used to follow-up mice parasitemia by diluting the sample 200-fold in HBSS buffer and assessing parasitemia under the VisiScope IT415 PH light microscope (VWR, United States). The rest of the collected blood was used to extract and purify the total gDNA using the DNeasy Blood & Tissue Kit (Qiagen, Germany) following the manufacturer’s guidelines. DNA samples were eluted in DNase/RNase-free water on equal volumes to the initial sample (i.e., no sample concentration). The resulting gDNA was used to evaluate the samples using the *Tev*PCR (used as a gold standard to assess positivity), Two-Pot *Tev*RPA-CRISPR and the One-Pot *Tev*RPA-CRISPR tests. The *Tev*PCR was performed as described in Claes et al. (2004). *Tev*RPA-CRISPR Two-Pot and the *Tev*RPA-CRISPR One-Pot tests were performed as described previously in this study on optimized conditions. Fluorescence values from positive and negative samples from the Two-Pot *Tev*RPA-CRISPR and the One-Pot *Tev*RPA-CRISPR tests were evaluated by a Receiver Operating Characteristic (ROC) curve analysis for determining test’s positivity thresholds, as well as sensitivity and specificity scores (Supplementary Table S2; Supplementary Figure S9).

### Ethics statement

All experiments, maintenance and care of the mice complied with the European Convention for the Protection of Vertebrate Animals (ECPVA) used for Experimental and Other Scientific Purposes guidelines (CETS No 123) and were approved by the Ethical Committee for Animal Experiments (ECAE) at the Vrije Universiteit Brussel (Permit Number: 17-220-02). Mice were monitored daily. Humane endpoints were used during the study, based on weight loss, whereby animals with >25% weight loss were sacrificed using carbon dioxide treatment. The study was conducted in accordance with the local legislation and institutional requirements.

### Statistical analysis

The GraphPad Prism 10 software was used for statistical analyses. Analytical sensitivity, and analytical specificity analyses were conducted using three technical replicates. The results presented were chosen as the most representative from two independent experiments. All statistical analyses were conducted using a one-way ANOVA, followed by Dunnett’s multiple comparison test. Values are expressed as mean ± standard deviation (SD) and *p-values* are shown. Specificity and sensitivity were evaluated through Receiver Operating Characteristic (ROC) curve analysis, which included a 95% confidence interval (CI) for both metrics, based on a sample size of n = 66.

## Results

### Development and Optimization of a *Tev*CRISPR-Cas12b assay for versatile and sensitive detection of *T*. *b*. *evansi*

Development of a *Tev*RPA-CRISPR test requires the pre-amplification of a double-stranded DNA (dsDNA) target by RPA, followed by a highly specific cleavage and detection of the resulting amplicon through the CRISPR-Cas12b machinery (Figure 1A). For this, it is critical to choose a proper target region for the Cas12b-sgRNA complex, which must contain a PAM sequence (5′-TTN-3′) followed by a protospacer sequence (Teng et al., 2018). The target sequence was chosen based on: (i) the presence within the *Tev*RPA amplicon, outside the primers or primer binding sites; (ii) the occurrence of a PAM sequence; (iii) the degree of nucleotide sequence identity between different *T. b. evansi* strains from different origins; and (iv) the absence of single nucleotide polymorphisms (SNPs).

First, the specificity of the RoTat 1.2 VSG gene to *T. b. evansi* type A was verified by PCR amplification (Supplementary Figure S1). Then, the *Tev*RPA targeted sequence included within the aDNAs collected from 13 *T. b. evansi* type A parasites was analyzed (Table 1). A 99.65% nucleotide sequence identity was observed, including the presence of a unique SNP in two geographically closely related *T. b. evansi* type A strains, being *T. b. evansi* KAZAKHSTAN and *T. b. evansi* STIB 816, from Kazakhstan and P.R. of China, respectively (Supplementary Figure S2). As a result, and in accordance with the above-mentioned criteria, six sgRNAs targeting the *Tev*RPA amplicon were designed (Supplementary Table S1). The efficacy of all sgRNAs was analyzed in conjunction with *Aap*Cas12b for cleaving (through *cis*-cleavage) and detecting (through *trans*-cleavage) *T. b. evansi* aDNA. Among the sgRNAs, sgRNA_6, designated as RoTat1.2sgRNA, exhibited the most rapid and robust fluorescence signals throughout the reaction (Supplementary Figure S3). Consequently, RoTat1.2sgRNA was selected for subsequent development stages of the *Tev*RPA-CRISPR diagnostic test. Next, the optimal *Aap*Cas12b:RoTat1.2sgRNA molar ratio to target *T. b. evansi* aDNA was evaluated, indicating a 4:1 ratio (i.e., 62.5 nM *Aap*Cas12b and 250 nM RoTat1.2sgRNA) as the best combination to reduce assay costs while maximizing the cleavage (Supplementary Figure S4). Previous studies have reported an optimal *Aap*Cas12b temperature range between 31°C and 59°C for the *cis*-cleavage and up to 50°C–60°C for the *trans*-cleavage (Joung et al., 2020; Teng et al., 2018; Huyke et al., 2022). To corroborate if those results apply to our CRISPR-*Aap*Cas12b- RoTat1.2sgRNA design, a temperature range analysis was performed for both *cis*- and *trans*-cleavage reactions. A nearly total *cis*-cleavage of the target *T. b. evansi* aDNA was observed up to 55°C, while at 60ºC–70°C the cleavage was reduced but still visible (Figure 1B). Nevertheless, the *trans*-cleavage of the fluorescent probes was optimal from 40ºC to 60°C, and progressively decreased but measurable when increasing the reaction temperature at 65ºC–70°C (Figure 1C). As such, 50°C was selected as the optimal reaction temperature. Finally, the analytical sensitivity of the *Tev*CRISPR-Cas12b assay was determined at the optimized reaction conditions. To achieve this, the *Tev*CRISPR-Cas12b assays were conducted on 1:10 serially diluted *T. b. evansi* aDNA samples, using a negative control sample in which aDNA was absent. Figure 1D shows that the *Tev*CRISPR-Cas12b assay detects target *T. b. evansi* aDNA up to a pM concentration.

### Integrated *Tev*RPA-CRISPR assay for highly sensitive and specific detection of *T*. *b*. *evansi*

**FIGURE 2:**
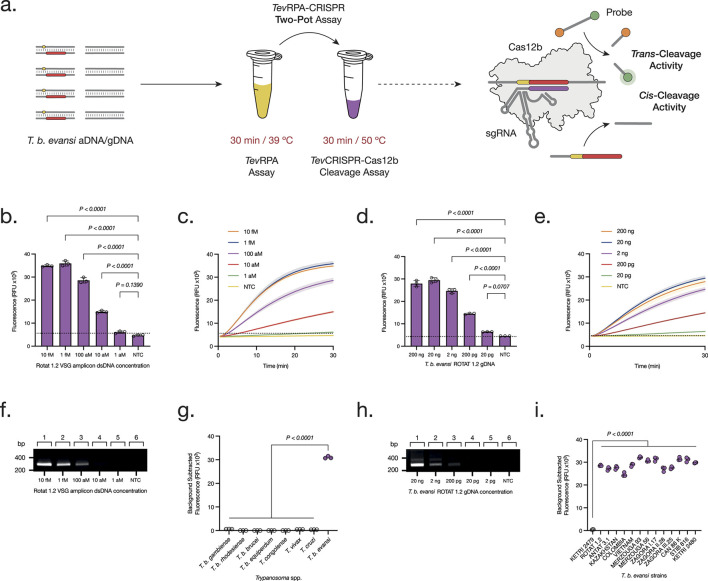
Development and Optimization of the Two-Pot *Tev*RPA-CRISPR test. **(A)** The Two-Pot *Tev*RPA-CRISPR assay is performed in two reactions, being *Tev*RPA for 30 min at 39°C, and *Tev*CRISPR-Cas12b for 30 min at 50°C. First, *T. b. evansi* aDNA or gDNA is amplified by the *Tev*RPA, and the resulting amplicons are recognized and *cis*-cleaved by the CRISPR-*Aap*Cas12b complex. This leads to the non-specific *trans*-cleavage of the FAM-Q probes, resulting in a measurable fluorescence signal of the released FAM reporters. **(B, D)** Analytical sensitivity assessment of the Two-Pot *Tev*RPA-CRISPR test to aDNA **(B)** and gDNA **(D)**. Background subtracted fluorescence of 3 technical replicates is plotted as mean ± standard deviation (SD). A Cut-off (No Template Control (NTC) mean +3SD) is indicated by the dashed line. **(C, E)** Kinetics of the *TevC*RISPR-Cas12b *trans*-cleavage from the analytical sensitivity assessment of the Two-Pot *Tev*RPA-CRISPR test to aDNA **(C)** and gDNA **(E)**. Fluorescence was measured over 30 min. Shaded regions represent SD of 3 technical replicates. **(F, H)** Analytical sensitivity assessment of the *Tev*RPA test to aDNA **(F)** and gDNA **(H)**. Lanes 1–5, serial 10-fold dilution of aDNA from 10 fM to 1 aM, and of gDNA from 20 ng to 2 pg; lane 6 NTC. Results were visualized on a 2% agarose gel pre-stained with ethidium bromide. **(G, I)** Analytical specificity assessment of the Two-Pot *Tev*RPA-CRISPR test to different *Trypanosoma* spp. **(G)** and *T. b. evansi* strains **(I)**. Background subtracted fluorescence of 3 technical replicates is plotted, where the mean is indicated as a dashed line. All statistical analyses were conducted using a one-way ANOVA, followed by Dunnett’s multiple comparison test. Significant differences between groups are denoted with the corresponding p-values listed above.

The *Aap*Cas12b-RoTat1.2sgRNA complex was designed with the aim of adapting this technology to the *Tev*RPA. Hence, first a Two-Pot test was developed, in which the RPA amplification is directly followed by the cleavage of the resulting amplicon through the CRISPR-Cas12b machinery, and the cleavage and detection of a fluorescent probe (Figure 2A). This test was performed following the optimal conditions for both reactions, in two separate tubes, with a total reaction time of 1 h. Using this approach, the analytical sensitivities of both *Tev*RPA and Two-Pot *Tev*RPA-CRISPR tests were evaluated. While *Tev*RPA allows to detect up to 100 aM of the target *T. b. evansi* aDNA, the Two-Pot *Tev*RPA-CRISPR test improved this detection limit by 10-fold, detecting up to 10 aM of *T. b. evansi* aDNA after 30 min of reaction (Figures 2B, C, F), or by 100-fold, detecting up to 1 aM of *T. b. evansi* aDNA after 60–120 min of reaction (Supplementary Figure S5). When using *T. b. evansi* gDNA instead, the *Tev*RPA allowed to detect up to 200 pg of the target gDNA, whereas the Two-Pot *Tev*RPA-CRISPR test improved this detection limit by 10-fold, detecting up to 20 pg of *T. b. evansi* gDNA after 30 min of reaction (Figures 2D, E, H), or by 100-fold, detecting up to 2 pg of *T. b. evansi* gDNA after 60–120 min of reaction (Supplementary Figure S6). Although the CRISPR-Cas12b technology enhances sensitivity, its primary advantage lies in ensuring test specificity by serving as a “second verification” of amplicon accuracy following the initial amplification step (Joung et al., 2020). To probe the test’s analytical specificity, the Two-Pot *Tev*RPA-CRISPR was performed on different *Trypanosoma* spp. gDNA samples (listed in Table 1) including those that can be found coexisting in the same territories as *T. b. evansi*. As expected, the *Tev*RPA-CRISPR test resulted in a positive fluorescence signal only when *T. b. evansi* type A gDNA was present (Figures 2G, I).

**FIGURE 3:**
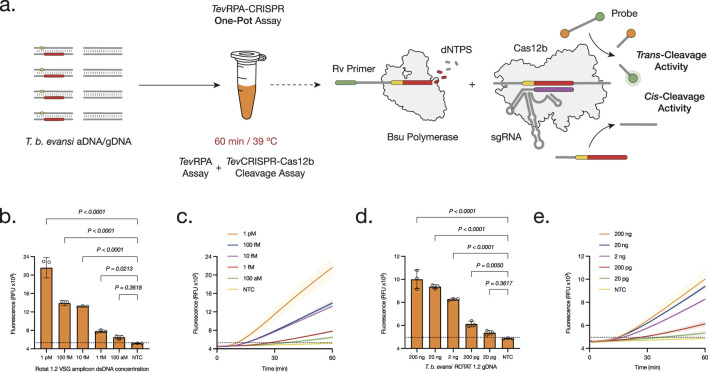
Development and Optimization of the One-Pot *Tev*RPA-CRISPR test. **(A)** The One-Pot *Tev*RPA-CRISPR assay is performed in one reaction for 60 min at 39°C. At the same time the amplicons from the *Tev*RPA are being synthesized, these are recognized and *cis*-cleaved by the CRISPR-*Aap*Cas12b complex. This leads to the non-specific *trans*-cleavage of the FAM-Q probes, resulting in a measurable fluorescence signal of the released FAM reporters. **(B, D)** Analytical sensitivity assessment of the One-Pot *Tev*RPA-CRISPR test to aDNA **(B)** and gDNA **(D)**. Background subtracted fluorescence of 3 technical replicates is plotted as mean ± standard deviation (SD). A Cut-off (No Template Control (NTC) mean+3SD) is indicated by the dashed line. **(C, E)** Kinetics of the analytical sensitivity assessment of the One-Pot *Tev*RPA-CRISPR test to aDNA **(C)** and gDNA **(E)**. Fluorescence was measured over 30 min. Shaded regions represent SD of 3 technical replicates. All statistical analyses were conducted using a one-way ANOVA, followed by Dunnett’s multiple comparison test. Significant differences between groups are denoted with the corresponding p-values listed above.

Having demonstrated the feasibility and adaptability of CRISPR-Cas12b within the *Tev*RPA Two-Pot system, combining both steps into a single-pot reaction was addressed, with the goal of creating an efficient and reliable diagnostic test for POC use. The One-Pot *Tev*RPA-CRISPR assay integrates RPA amplification with CRISPR-Cas12b-mediated cleavage and detection within a single reaction mixture, enabling the detection of *T. b. evansi* type A gDNA within 1 h. The amplification rate and efficiency of these RPA-CRISPR assays are significantly affected by primer concentration and magnesium acetate (MgOAc) levels. Therefore, the optimized concentration for both reagents was determined, being 14 mM MgOAc and 480 mM *Tev*RPA primers, respectively (Supplementary Figure S7). Finally, the analytical sensitivities of the One-Pot *Tev*RPA-CRISPR test were evaluated, detecting up to 100–10 aM of *T. b. evansi* aDNA (Figures 3B, C; Supplementary Figure S8), and 20 pg of *T. b. evansi* gDNA (Figures 3D, E; Supplementary Figure S8).

### *Tev*RPA-CRISPR assays detect active *T*. *b*. *evansi* infections and Cure with PCR-Level accuracy in experimental mouse models

**FIGURE 4:**
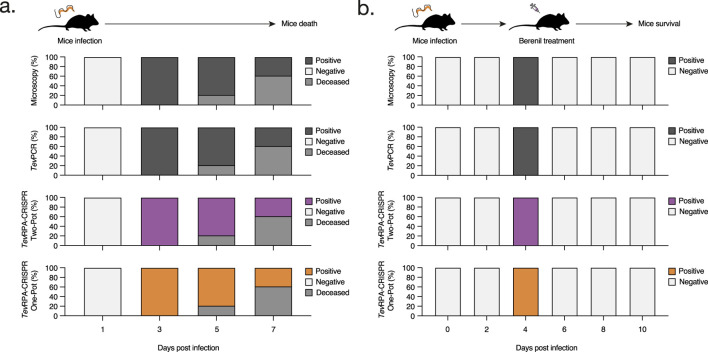
Assessment of the *Tev*RPA-CRISPR assays to detect both active and cured *T. b. evansi* infections. **(A)** C57BL/6 mice were infected with *T. b. evansi* STIB 816 (n = 5) and the presence of parasites was monitored over the course of the infection by microscopy, *Tev*PCR, Two-Pot *Tev*RPA-CRISPR and One-Pot *Tev*RPA-CRISPR. The results are showed as the percentages of mice that scored positive or negative at the above-mentioned techniques. **(B)** C57BL/6 mice infected with *T. b. evansi* STIB 816 (n = 5) were treated with Berenil at 5 days post-infection. The presence of parasites was followed by microscopy, *Tev*PCR, Two-Pot *Tev*RPA-CRISPR and One-Pot *Tev*RPA-CRISPR along the experiment. The panels and color codes are identical to those used in panel **(A)**. The *Tev*PCR, Two-Pot *Tev*RPA-CRISPR, and One-Pot *Tev*RPA-CRISPR read-outs are shown in Figure 5. These results were selected as the most representative from two independent experiments.

**FIGURE 5:**
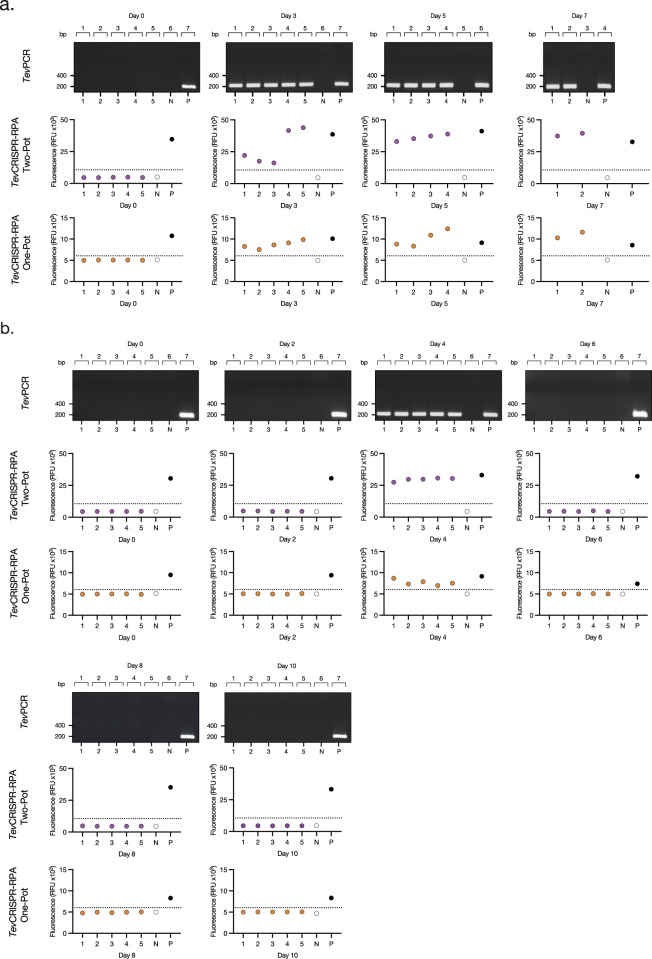
Read-outs from the Assessment of the *Tev*RPA-CRISPR performance on experimental *T. b. evansi* infections. **(A)** *Tev*PCR, Two-Pot *Tev*RPA-CRISPR and One-Pot *Tev*RPA-CRISPR results from the mouse infection of Figure 4A. **(B)** *Tev*PCR, Two-Pot *Tev*RPA-CRISPR, and One-Pot *Tev*RPA-CRISPR results from the mouse infection of Figure 4B. Numbers from 1–5 below the plots correspond to each of the individual mice analyzed per group. N and P correspond to the negative control (water only), and the positive control (*T. b. evansi* STIB 816 gDNA). *Tev*PCR results were visualized on a 2% agarose gel pre-stained with ethidium bromide. Fluorescence values from the Two-Pot *Tev*RPA-CRISPR and One-Pot *Tev*RPA-CRISPR results were evaluated by a Receiver Operating Characteristic (ROC) curve analysis to determine the test’s positivity thresholds (dashed lines).

After developing the Two-Pot and One-Pot *Tev*RPA-CRISPR designs, test efficacy in diagnosing both active and cured infections of *T. b. evansi* was validated. Ten C57BL/6 mice were infected with *T. b. evansi* STIB 816, divided into two groups. The presence of parasites was assessed by microscopy, *Tev*PCR (Claes et al., 2004), Two-pot *Tev*RPA-CRISPR, and One-Pot *Tev*RPA-CRISPR at various time points of infection. Group 1 was left untreated, while Group 2 was treated with Berenil 5 days post-infection (Figures 4A, B). Both Two-Pot and One-Pot *Tev*RPA-CRISPR assays accurately detected all infected samples in both untreated and treated groups, matching the performance of the gold standard *Tev*PCR (Kappa value = 1) (Figures 5A, B). All infected mice in Group 1 were euthanized by day 8 post-infection, as they started to show signs of infection-associated pathology. In contrast, all mice in Group 2 survived, indicating successful parasite clearance following Berenil treatment. Both Two-Pot and One-Pot *Tev*RPA-CRISPR yielded negative results in post-treatment non-infected samples, as corroborated by *Tev*PCR, validating their effectiveness as “test-of-cure” assays (Figure 5B). Finally, the preliminary sensitivity and specificity of both *Tev*RPA-CRISPR tests were evaluated, achieving 100% for both metrics (Supplementary Table S2).

## Discussion

In this study, we developed and optimized a *Tev*RPA-CRISPR assay to be used as a highly specific and sensitive alternative for POC/PON diagnosis of *T*. *b*. *evansi* active infections. This assay integrates an RPA for target amplification together with a CRISPR-Cas12b system for amplicon detection. Target amplification is facilitated by a *Tev*RPA, which specifically targets the RoTat 1.2 VSG gene unique to *T*. *b*. *evansi* type A parasites (Verloo et al., 2001). As demonstrated in this study, the nucleotide sequence of the RoTat 1.2 VSG region is highly conserved across *T. b. evansi* type A strains from various origins, ensuring a broad applicability of the test. Subsequently, the amplified target detection is carried out by the *Tev*CRISPR-Cas12b cleavage assay, which combines the CRISPR-*Aap*Cas12b together with the RoTat1.2sgRNA, forming the CRISPR complex. Our findings reveal that the *Tev*CRISPR-Cas12b cleavage assay can reliably detect picomolar concentrations of the target *T. b. evansi* aDNA, consistent with the reported analytical sensitivities for CRISPR-*Aap*Cas12b systems (Huyke et al., 2022). Despite being a highly sensitive assay, the pre-amplification of the target DNA is still recommended when directly detecting gDNA samples. Besides its low limit of detection, the CRISPR-*Aap*Cas12b system has been reported to exhibit minimal to no off-target cis-cleavage activity (Teng et al., 2018; Liu et al., 2017). This quality makes it highly specific and adaptable to any amplification method when applied as a second-step reaction (i.e., Two-Pot system), serving as a reliable second result verification on inconclusive *T. b. evansi* tests. The CRISPR-*Aap*Cas12b system also proved to be robust and optimally operate in a wide range of reaction temperatures (40°C to 60°C), as already reported in other studies (Joung et al., 2020; Teng et al., 2018; Huyke et al., 2022; Nguyen et al., 2022). This quality makes it compatible and highly adaptable to most isothermal nucleic acid amplification reactions if integrated into a single-step reaction (i.e., One-Pot system) (Sereno et al., 2022).

The combined test approach we explored in this study utilizes our previously established *Tev*RPA assay, which exhibits high specificity for *T. b. evansi* type A and integrates it with the *Tev*CRISPR-Cas12b cleavage assay. While many researchers choose to adapt RPA to other Cas proteins, like Cas13 or Cas12a, it has been proposed that when combined with RPA, Cas12b, and specifically *Aap*Cas12b yields a better performance (Aman et al., 2021). Our data shows that the Two-Pot *Tev*RPA-CRISPR assay substantially enhances analytical sensitivity by a factor of up to 100 compared to the traditional *Tev*RPA method, while also exhibiting robust analytical specificity with no cross-reactivity to other *Trypanosoma* species (Table 3). This assay provides an optimal solution for detecting *T. b. evansi* type A parasites in both laboratory and field settings. Although *Tev*RPA offers a rapid alternative to the gold-standard *Tev*PCR in laboratory settings, it is prone to non-specific amplification leading to false positive results. The Two-Pot *Tev*RPA-CRISPR assay overcomes this limitation, providing a more reliable and precise diagnostic tool. In field settings, the Two-Pot *Tev*RPA-CRISPR facilitates the initial fast and user-friendly screening with *Tev*RPA, while follow-up laboratory-based confirmation and detailed analysis with the *Tev*CRISPR-Cas12b assay ensures an accurate and reliable result.

**TABLE 3:** Intrinsic properties of Two-Pot *Tev*RPA-CRISPR and One-Pot *Tev*RPA-CRISPR. Specificity and sensitivity were assessed using Receiver Operating Characteristic (ROC) curve analysis (see Supplementary Figure S9). The results include a 95% confidence interval for both metrics, calculated from a sample size of n = 66.

	*Analytical Specificity*	*Analytical Sensitivity*	*Specificity*	*Sensitivity*
*Tev*CRISPR-RPA Two-Pot	*T. evansi* type A	10-1 aM aDNA 20-2 pg gDNA	100 % (95% CI: 91.24–100%)	100 % (95% CI: 87.13–100%)
*Tev*CRISPR-RPA One-Pot	*T. evansi* type A	100-10 aM aDNA 20 pg gDNA	100 % (95% CI: 91.24–100%)	100 % (95% CI: 87.13–100%)

Integrating the *Tev*RPA and *Tev*CRISPR-Cas12b assays into a single reaction simplifies the workflow while preserving high analytical sensitivity and specificity. The One-Pot *Tev*RPA-CRISPR assay achieved analytical sensitivities comparable to the Two-Pot *Tev*RPA-CRISPR assay, while maintaining a specific detection of *T. b. evansi* type A parasites (Table 3). The One-Pot *Tev*RPA-CRISPR assay, while well-suited for laboratory settings, was designed to meet the need for a user-friendly yet sensitive and specific test suitable for POC/PON diagnosis in field settings. In fact, the One-Pot *Tev*RPA-CRISPR can be carried out using a body heater or portable water bath, and the results can be observed using cost-effective blue-light transilluminators (Supplementary Figure S10), powered by batteries or connected to a mobile phone, as well as through standard lateral-flow devices, which require no additional equipment (Myhrvold et al., 2018; Deng et al., 2023). Accordingly, this assay offers a compelling alternative to standard screening tools, like CATT/*T. b. evansi* or ELISA/*T. b. evansi*.

Diagnostic accuracy evaluation of the newly developed Two-Pot and One-Pot *Tev*RPA-CRISPR assays was done in a setting that compared results of both active and cured *T. b. evansi* infections, using an experimental mouse model. Both assays showed full concordance with the gold standard *Tev*PCR, achieving 100% sensitivity and specificity. This performance confirms the potential for effectively monitoring treatment efficacy and parasite clearance, establishing both *Tev*RPA-CRISPR assays as valuable tools for managing *T. b. evansi* infections.

In conclusion, our findings demonstrate that the newly developed *Tev*RPA-CRISPR assays offer a robust and reliable proof-of-concept, with significant potential as viable alternatives to current screening tools for both laboratory and field settings. Future efforts now must focus on extensive field trials and possibly further optimization, to ensure assay performance and applicability in a POC setting.

## Data Availability

The datasets presented in this study can be found in online repositories. The names of the repository/repositories and accession number(s) can be found in the article/Supplementary Material.
