# Supplementary material for: A CRISPR-Cas-based recombinase polymerase amplification assay for ultra-sensitive detection of active Trypanosoma brucei evansi infections
Source: Front Mol Biosci. 2025 Feb 14;12:1512970. doi: 10.3389/fmolb.2025.1512970 (PMC11867955; doi:10.3389/fmolb.2025.1512970)
Supplement: Supplementary file 1 [file DataSheet1.pdf]

Supplementary Material

	RNA sequence (5'-3')
**sgRNA_1**	GUCUAGAGGACAGAAUUUUUCAACGGGUGUGCCAAUGGCCACUUUCCAGGUGGCAAA GCCCGUUGAGCUUCUCAAAUCUGAGAAGUGGCAC**CUAAAGCACGCGGUUGGCAA**
**sgRNA_2**	GUCUAGAGGACAGAAUUUUUCAACGGGUGUGCCAAUGGCCACUUUCCAGGUGGCAAA GCCCGUUGAGCUUCUCAAAUCUGAGAAGUGGCAC**UAAAGCACGCGGUUGGCAAC**
**sgRNA_3**	GUCUAGAGGACAGAAUUUUUCAACGGGUGUGCCAAUGGCCACUUUCCAGGUGGCAAA GCCCGUUGAGCUUCUCAAAUCUGAGAAGUGGCAC**GCAACAGCAAGUUUUGUGUG**
**sgRNA_4**	GUCUAGAGGACAGAAUUUUUCAACGGGUGUGCCAAUGGCCACUUUCCAGGUGGCAAA GCCCGUUGAGCUUCUCAAAUCUGAGAAGUGGCAC**UGUGUGGGCAAAGCCGACGG**
**sgRNA_5**	GUCUAGAGGACAGAAUUUUUCAACGGGUGUGCCAAUGGCCACUUUCCAGGUGGCAAA GCCCGUUGAGCUUCUCAAAUCUGAGAAGUGGCAC**GUGUGGGCAAAGCCGACGGC**
**sgRNA_6**	GUCUAGAGGACAGAAUUUUUCAACGGGUGUGCCAAUGGCCACUUUCCAGGUGGCAAA GCCCGUUGAGCUUCUCAAAUCUGAGAAGUGGCAC**UGUGGGCAAAGCCGACGGCA**
**Scaffold sgRNA**	GUCUAGAGGACAGAAUUUUUCAACGGGUGUGCCAAUGGCCACUUUCCAGGUGGCAAA GCCCGUUGAGCUUCUCAAAUCUGAGAAGUGGCAC

Supplementary Table 1. Scaffold sgRNA and sgRNAs employed in this study.

	Threshold	*Sensitivity (%)*	*95% CI*	*Specificity (%)*	*95% CI*
**One-pot**	> 6.050	100	87.13% to 100%	100	91.24% to 100%
**Two-pot**	> 10.62	100	87.13% to 100%	100	91.24% to 100%

Supplementary Table 2. Two-Pot *Tev*RPA-CRISPR and One-Pot *Tev*RPA-CRISPR tests positivity thresholds, as well as sensitivity and specificity scores. Evaluated by a Receiver Operating Characteristic (ROC) curve analysis (See Figure S9).

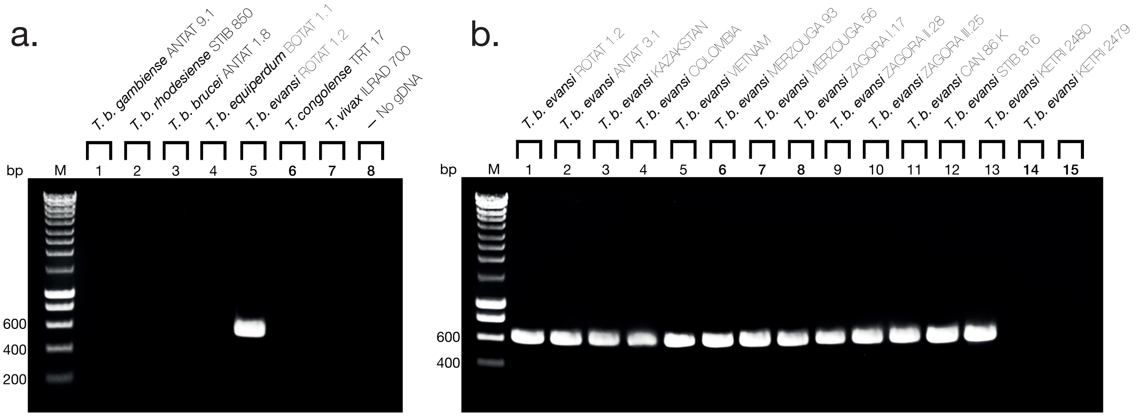

Supplementary Figure 2. Verification of the specificity of the RoTat 1.2 VSG gene to *T. b. evansi* type A. (a) Lane 1, *T. b. gambiense* ANTAT 9.1, lane 2, *T. b. rhodesiense* STIB 850, lane 3, *T. b. brucei* ANTAT 1.8, lane 4, *T. b. equiperdum* BOTAT 1.1, lane 5, *T. b. evansi* ROTAT 1.2, lane 6, *T. congolense* TRT 17, lane 7, *T. vivax* ILRAD 700, lane 8, NTC. (b) Lane 1, *T. b. evansi* ROTAT 1.2, lane 2, *T. b. evansi* ANTAT 3.1, lane 3, *T. b. evansi* KAZAKHSTAN, lane 4, *T. b. evansi* COLOMBIA, lane 5, *T. b. evansi* VIETNAM, lane 6, *T. b. evansi* MERZOUGA 93, lane 7, *T. b. evansi* MERZOUGA 56, lane 8, *T. b. evansi* ZAGORA I.17, lane 9, *T. b. evansi* ZAGORA II.28, lane 10, *T. b. evansi* ZAGORA III.25, lane 11, *T. b. evansi* CAN 86 K, lane 12, *T. b. evansi* STIB 816, lane 13, *T. b. evansi* KETRI 2480, lane 14, *T. b. evansi* KETRI 2479, lane 15, NTC. Results were visualized on 2% agarose gel pre-stained with ethidium bromide.

ROTAT 1.2 RPA FW primer binding site **99.65% nucleotide sequence identity**

50

CACCGAAGCAAGCGCAGCAAGAGGGTTAGCAGCGGCCGCAGATAGACACATACGAGCAGC 60 *T. b. evansi* ROTAT 1.2
CACCGAAGCAAGCGCAGCAAGAGGGTTAGCAGCGGCCGCAGATAGACACATACGAGCAGC 60 *T. b. evansi* ANTAT 1.3
CACCGAAGCAAGCGCAGCAAGAGGGTTAGCAGCGGCCGCAGATAGACACATACGAGCAGC 60 *T. b. evansi* COLOMBIA
CACCGAAGCAAGCGCAGCAAGAGGGTTAGCAGCGGCCGCAGATAGACACATACGAGCAGC 60 *T. b. evansi* VIETNAM
CACCGAAGCAAGCGCAGCAAGAGGGTTAGCAGCGGCCGCAGATAGACACATACGAGCAGC 60 *T. b. evansi* MERZOUGA 93
CACCGAAGCAAGCGCAGCAAGAGGGTTAGCAGCGGCCGCAGATAGACACATACGAGCAGC 60 *T. b. evansi* MERZOUGA 56
CACCGAAGCAAGCGCAGCAAGAGGGTTAGCAGCGGCCGCAGATAGACACATACGAGCAGC 60 *T. b. evansi* ZAGORA I.17
CACCGAAGCAAGCGCAGCAAGAGGGTTAGCAGCGGCCGCAGATAGACACATACGAGCAGC 60 *T. b. evansi* ZAGORA II.28
CACCGAAGCAAGCGCAGCAAGAGGGTTAGCAGCGGCCGCAGATAGACACATACGAGCAGC 60 *T. b. evansi* ZAGORA III.25
CACCGAAGCAAGCGCAGCAAGAGGGTTAGCAGCGGCCGCAGATAGACACATACGAGCAGC 60 *T. b. evansi* CAN 86 K
CACCGAAGCAAGCGCAGCAAGAGGGTTAGCAGCGGCCGCAGATAGACACATACGAGCAGC 60 *T. b. evansi* KETRI 2480
CACCGAAGCAAGCGCAGCAAGAGGGTTAGCAGCGGCCGCAGATAGACACGTACGAGCAGC 60 *T. b. evansi* KAZAKSTAN
CACCGAAGCAAGCGCAGCAAGAGGGTTAGCAGCGGCCGCAGATAGACACGTACGAGCAGC 60 *T. b. evansi* STIB 816
*** **********

GGCCACCACGGCGAAAGACAAAAGCAGGGTAATTCTGCCCGCAGTTGCCTATGGCGGCGA 120 *T. b. evansi* ROTAT 1.2
GGCCACCACGGCGAAAGACAAAAGCAGGGTAATTCTGCCCGCAGTTGCCTATGGCGGCGA 120 *T. b. evansi* ANTAT 1.3
GGCCACCACGGCGAAAGACAAAAGCAGGGTAATTCTGCCCGCAGTTGCCTATGGCGGCGA 120 *T. b. evansi* COLOMBIA
GGCCACCACGGCGAAAGACAAAAGCAGGGTAATTCTGCCCGCAGTTGCCTATGGCGGCGA 120 *T. b. evansi* VIETNAM
GGCCACCACGGCGAAAGACAAAAGCAGGGTAATTCTGCCCGCAGTTGCCTATGGCGGCGA 120 *T. b. evansi* MERZOUGA 93
GGCCACCACGGCGAAAGACAAAAGCAGGGTAATTCTGCCCGCAGTTGCCTATGGCGGCGA 120 *T. b. evansi* MERZOUGA 56
GGCCACCACGGCGAAAGACAAAAGCAGGGTAATTCTGCCCGCAGTTGCCTATGGCGGCGA 120 *T. b. evansi* ZAGORA I.17
GGCCACCACGGCGAAAGACAAAAGCAGGGTAATTCTGCCCGCAGTTGCCTATGGCGGCGA 120 *T. b. evansi* ZAGORA II.28
GGCCACCACGGCGAAAGACAAAAGCAGGGTAATTCTGCCCGCAGTTGCCTATGGCGGCGA 120 *T. b. evansi* ZAGORA III.25
GGCCACCACGGCGAAAGACAAAAGCAGGGTAATTCTGCCCGCAGTTGCCTATGGCGGCGA 120 *T. b. evansi* CAN 86 K
GGCCACCACGGCGAAAGACAAAAGCAGGGTAATTCTGCCCGCAGTTGCCTATGGCGGCGA 120 *T. b. evansi* KETRI 2480
GGCCACCACGGCGAAAGACAAAAGCAGGGTAATTCTGCCCGCAGTTGCCTATGGCGGCGA 120 *T. b. evansi* KAZAKSTAN
GGCCACCACGGCGAAAGACAAAAGCAGGGTAATTCTGCCCGCAGTTGCCTATGGCGGCGA 120 *T. b. evansi* STIB 816
****

AGTCGCAGGGGCGATTTCATCGGCGCTAAAATTTCTAAAGCACGCGGTTGGCAACAGCAA 180 *T. b. evansi* ROTAT 1.2
AGTCGCAGGGGCGATTTCATCGGCGCTAAAATTTCTAAAGCACGCGGTTGGCAACAGCAA 180 *T. b. evansi* ANTAT 1.3
AGTCGCAGGGGCGATTTCATCGGCGCTAAAATTTCTAAAGCACGCGGTTGGCAACAGCAA 180 *T. b. evansi* COLOMBIA
AGTCGCAGGGGCGATTTCATCGGCGCTAAAATTTCTAAAGCACGCGGTTGGCAACAGCAA 180 *T. b. evansi* VIETNAM
AGTCGCAGGGGCGATTTCATCGGCGCTAAAATTTCTAAAGCACGCGGTTGGCAACAGCAA 180 *T. b. evansi* MERZOUGA 93
AGTCGCAGGGGCGATTTCATCGGCGCTAAAATTTCTAAAGCACGCGGTTGGCAACAGCAA 180 *T. b. evansi* MERZOUGA 56
AGTCGCAGGGGCGATTTCATCGGCGCTAAAATTTCTAAAGCACGCGGTTGGCAACAGCAA 180 *T. b. evansi* ZAGORA I.17
AGTCGCAGGGGCGATTTCATCGGCGCTAAAATTTCTAAAGCACGCGGTTGGCAACAGCAA 180 *T. b. evansi* ZAGORA II.28
AGTCGCAGGGGCGATTTCATCGGCGCTAAAATTTCTAAAGCACGCGGTTGGCAACAGCAA 180 *T. b. evansi* ZAGORA III.25
AGTCGCAGGGGCGATTTCATCGGCGCTAAAATTTCTAAAGCACGCGGTTGGCAACAGCAA 180 *T. b. evansi* CAN 86 K
AGTCGCAGGGGCGATTTCATCGGCGCTAAAATTTCTAAAGCACGCGGTTGGCAACAGCAA 180 *T. b. evansi* KETRI 2480
AGTCGCAGGGGCGATTTCATCGGCGCTAAAATTTCTAAAGCACGCGGTTGGCAACAGCAA 180 *T. b. evansi* KAZAKSTAN
AGTCGCAGGGGCGATTTCATCGGCGCTAAAATTTCTAAAGCACGCGGTTGGCAACAGCAA 180 *T. b. evansi* STIB 816
****

GTTTTGTGTGGGCAAAGCCGACGGCACAAATGCCGACGGTAACAACGAAATCGACGCGCT 240 *T. b. evansi* ROTAT 1.2
GTTTTGTGTGGGCAAAGCCGACGGCACAAATGCCGACGGTAACAACGAAATCGACGCGCT 240 *T. b. evansi* ANTAT 1.3
GTTTTGTGTGGGCAAAGCCGACGGCACAAATGCCGACGGTAACAACGAAATCGACGCGCT 240 *T. b. evansi* COLOMBIA
GTTTTGTGTGGGCAAAGCCGACGGCACAAATGCCGACGGTAACAACGAAATCGACGCGCT 240 *T. b. evansi* VIETNAM
GTTTTGTGTGGGCAAAGCCGACGGCACAAATGCCGACGGTAACAACGAAATCGACGCGCT 240 *T. b. evansi* MERZOUGA 93
GTTTTGTGTGGGCAAAGCCGACGGCACAAATGCCGACGGTAACAACGAAATCGACGCGCT 240 *T. b. evansi* MERZOUGA 56
GTTTTGTGTGGGCAAAGCCGACGGCACAAATGCCGACGGTAACAACGAAATCGACGCGCT 240 *T. b. evansi* ZAGORA I.17
GTTTTGTGTGGGCAAAGCCGACGGCACAAATGCCGACGGTAACAACGAAATCGACGCGCT 240 *T. b. evansi* ZAGORA II.28
GTTTTGTGTGGGCAAAGCCGACGGCACAAATGCCGACGGTAACAACGAAATCGACGCGCT 240 *T. b. evansi* ZAGORA III.25
GTTTTGTGTGGGCAAAGCCGACGGCACAAATGCCGACGGTAACAACGAAATCGACGCGCT 240 *T. b. evansi* CAN 86 K
GTTTTGTGTGGGCAAAGCCGACGGCACAAATGCCGACGGTAACAACGAAATCGACGCGCT 240 *T. b. evansi* KETRI 2480
GTTTTGTGTGGGCAAAGCCGACGGCACAAATGCCGACGGTAACAACGAAATCGACGCGCT 240 *T. b. evansi* KAZAKSTAN
GTTTTGTGTGGGCAAAGCCGACGGCACAAATGCCGACGGTAACAACGAAATCGACGCGCT 240 *T. b. evansi* STIB 816
****

AGGGTGCGGCGAAGCCAA**CTATGACACCTCGGCCCCAGGAGACAGCTAC** 289 *T. b. evansi* ROTAT 1.2
AGGGTGCGGCGAAGCCAA**CTATGACACCTCGGCCCCAGGAGACAGCTAC** 289 *T. b. evansi* ANTAT 1.3
AGGGTGCGGCGAAGCCAA**CTATGACACCTCGGCCCCAGGAGACAGCTAC** 289 *T. b. evansi* COLOMBIA
AGGGTGCGGCGAAGCCAA**CTATGACACCTCGGCCCCAGGAGACAGCTAC** 289 *T. b. evansi* VIETNAM
AGGGTGCGGCGAAGCCAA**CTATGACACCTCGGCCCCAGGAGACAGCTAC** 289 *T. b. evansi* MERZOUGA 93
AGGGTGCGGCGAAGCCAA**CTATGACACCTCGGCCCCAGGAGACAGCTAC** 289 *T. b. evansi* MERZOUGA 56
AGGGTGCGGCGAAGCCAA**CTATGACACCTCGGCCCCAGGAGACAGCTAC** 289 *T. b. evansi* ZAGORA I.17
AGGGTGCGGCGAAGCCAA**CTATGACACCTCGGCCCCAGGAGACAGCTAC** 289 *T. b. evansi* ZAGORA II.28
AGGGTGCGGCGAAGCCAA**CTATGACACCTCGGCCCCAGGAGACAGCTAC** 289 *T. b. evansi* ZAGORA III.25
AGGGTGCGGCGAAGCCAA**CTATGACACCTCGGCCCCAGGAGACAGCTAC** 289 *T. b. evansi* CAN 86 K
AGGGTGCGGCGAAGCCAA**CTATGACACCTCGGCCCCAGGAGACAGCTAC** 289 *T. b. evansi* KETRI 2480
AGGGTGCGGCGAAGCCAA**CTATGACACCTCGGCCCCAGGAGACAGCTAC** 289 *T. b. evansi* KAZAKSTAN
AGGGTGCGGCGAAGCCAA**CTATGACACCTCGGCCCCAGGAGACAGCTAC** 289 *T. b. evansi* STIB 816
****

ROTAT 1.2 RPA RV primer binding site

Supplementary Figure 2. Assessment of the nucleotide sequence identity between different *T. b. evansi* type A strains. *Tev*RPA FW and RV primer binding sites are highlighted in bold. A single SNP is identified and labelled at position 50.

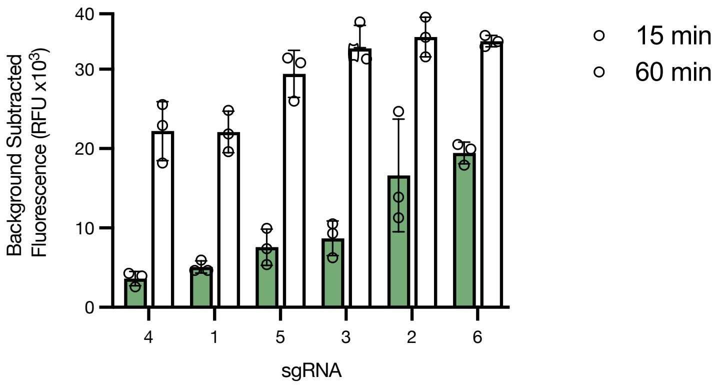

Supplementary Figure 3. Assessment of the best performing sgRNAs at the *Tev*CRISPR-Cas12b *trans*-cleavage assay. Background subtracted fluorescence of 3 technical replicates is plotted as mean ± standard deviation (SD). Measurements were taken after 15 (green colored bars) and 60 (white colored bars) min of reaction.

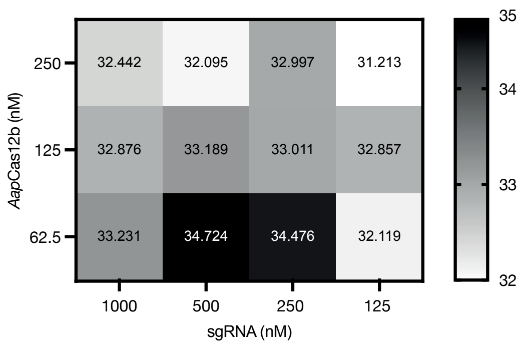

Supplementary Figure 4. Determination of the optimal *Aap*Cas12b:RoTat1.2sgRNA molar ratios at the *Tev*CRISPR-Cas12b *trans*-cleavage assay. Heatmap displays mean background subtracted fluorescence values of 3 technical replicates. Measurements were taken after 60 min of reaction.

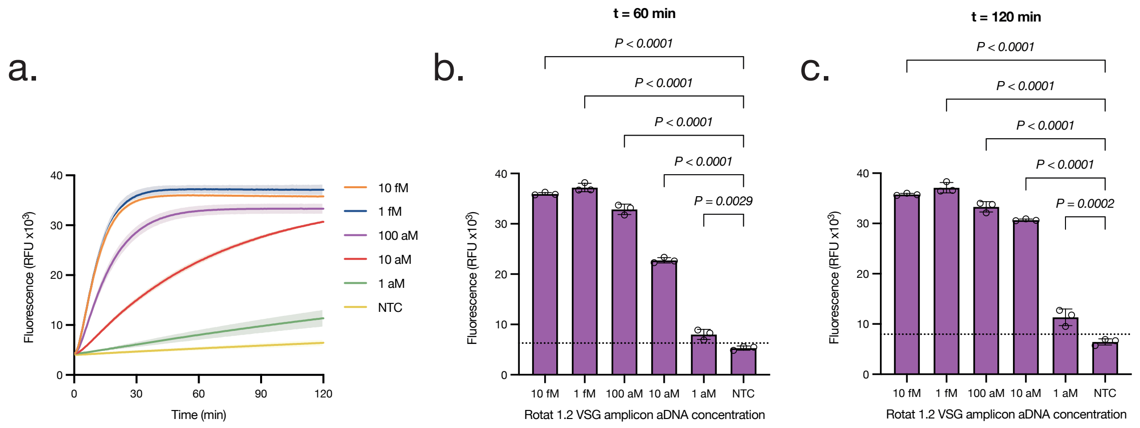

Supplementary Figure 5. (a) Kinetics of the *Tev*CRISPR-Cas12b *trans*-cleavage from the analytical sensitivity assessment of the Two-Pot *Tev*RPA-CRISPR test to aDNA. Fluorescence was measured over 120 min. Shaded regions represent SD of 3 technical replicates. (b)(c) Analytical sensitivity assessment of the Two-Pot *Tev*RPA-CRISPR test to aDNA at 60 and 120 min of reaction. Background subtracted fluorescence of 3 technical replicates is plotted as mean ± standard deviation (SD). A Cut-off (No Template Control (NTC) mean + 3 times the SD) is indicated by the dashed line. All statistical analyses were conducted using a one-way ANOVA, followed by a Dunnett's multiple comparison test. Significant differences between groups are denoted with the corresponding p-values listed above.

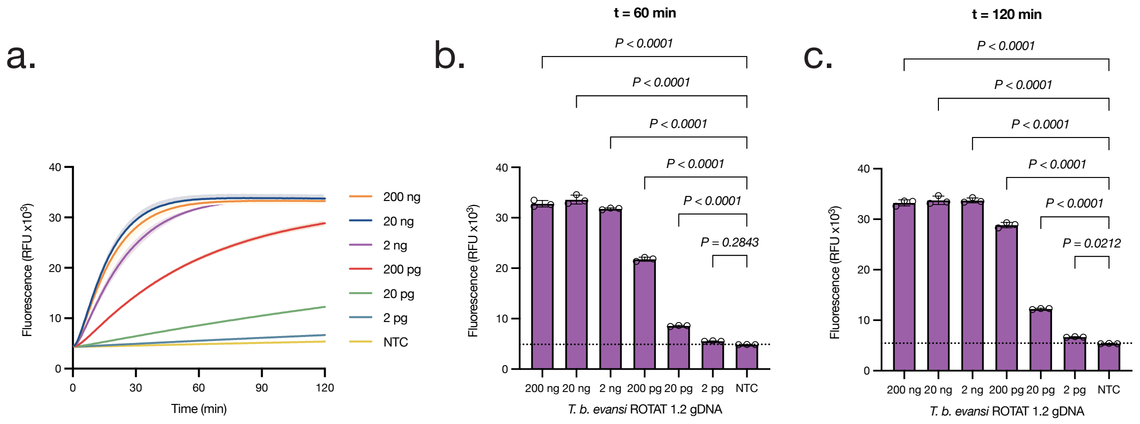

Supplementary Figure 6. (a) Kinetics of the *Tev*CRISPR-Cas12b *trans*-cleavage from the analytical sensitivity assessment of the Two-Pot *Tev*RPA-CRISPR test to gDNA. Fluorescence was measured over 120 min. Shaded regions represent SD of 3 technical replicates. (b)(c) Analytical sensitivity assessment of the Two-Pot *Tev*RPA-CRISPR test to gDNA at 60 and 120 min of reaction. Background subtracted fluorescence of 3 technical replicates is plotted as mean ± standard deviation (SD). A Cut-off (No Template Control (NTC) mean + 3 times the SD) is indicated by the dashed line. All statistical analyses were conducted using a one-way ANOVA, followed by a Dunnett's multiple comparison test. Significant differences between groups are denoted with the corresponding p-values listed above.

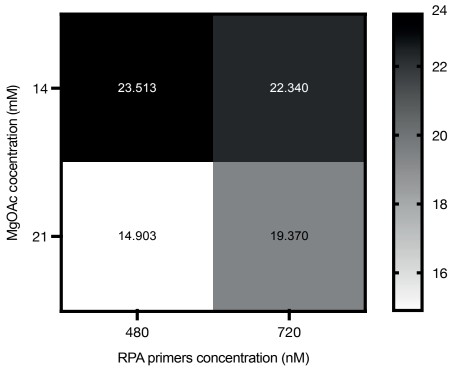

Supplementary Figure 7. Determination of the optimal MgOAc:RPA primers molar concentrations at the One-Pot *Tev*RPA-CRISPR test. Heatmap displays mean background subtracted fluorescence values of 3 technical replicates. Measurements were taken after 120 min of reaction.

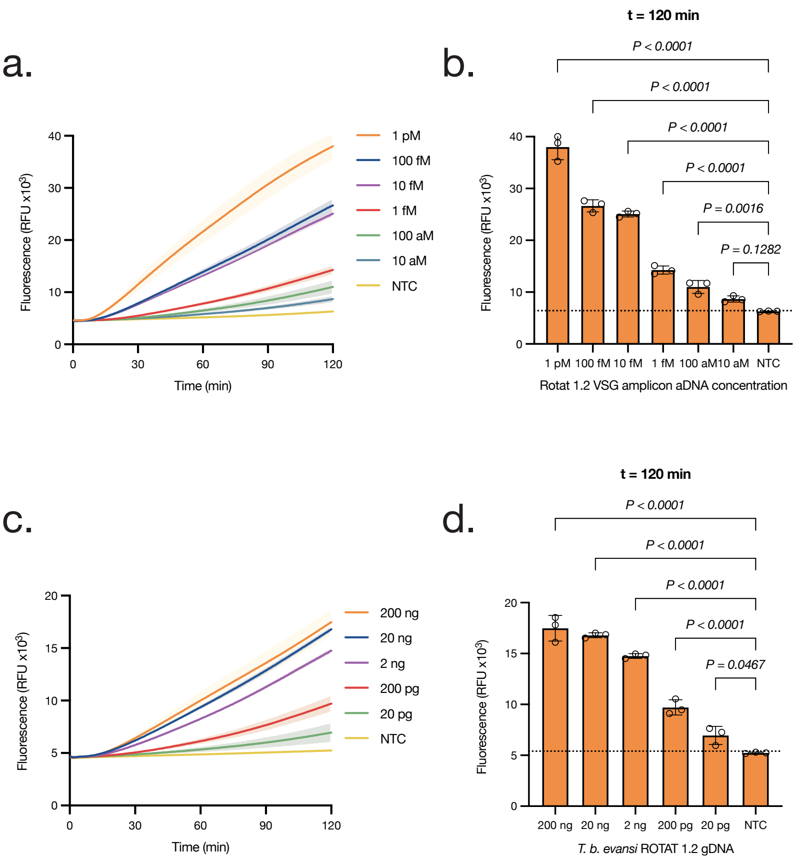

Supplementary Figure 8. (a)(c) Kinetics of the *Tev*CRISPR-Cas12b *trans*-cleavage from the analytical sensitivity assessment of the One-Pot *Tev*RPA-CRISPR test to aDNA and gDNA. Fluorescence was measured over 120 min. Shaded regions represent SD of 3 technical replicates. (b)(c) Analytical sensitivity assessment of the One-Pot *Tev*RPA-CRISPR test to aDNA and gDNA at 120 min of reaction. Background subtracted fluorescence of 3 technical replicates is plotted as mean ± standard deviation (SD). A Cut-off (No Template Control (NTC) mean + 3 times the SD) is indicated by the dashed line. All statistical analyses were conducted using a one-way ANOVA, followed by a Dunnett's multiple comparison test. Significant differences between groups are denoted with the corresponding p-values listed above.

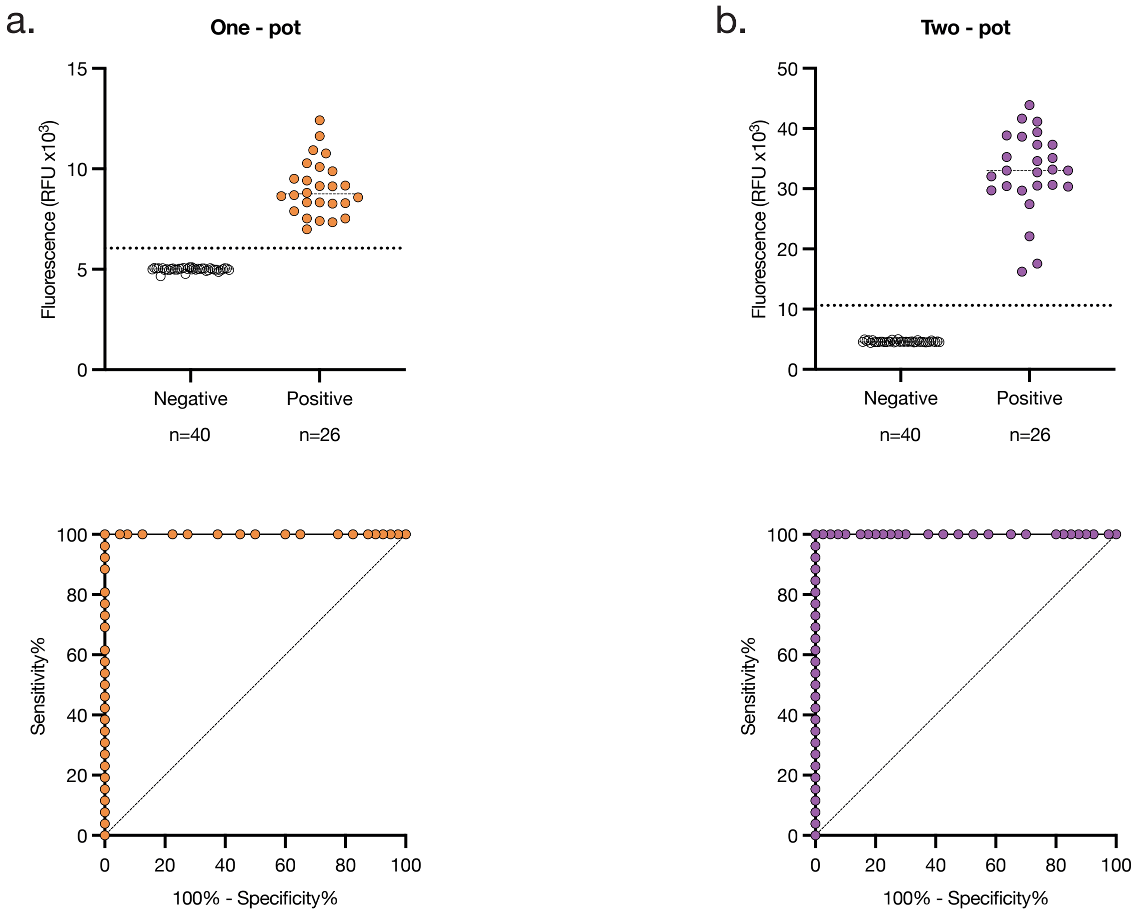

Supplementary Figure 9. (a) Two-Pot *Tev*RPA-CRISPR and (b) One-Pot *Tev*RPA-CRISPR tests positivity thresholds, as well as a Receiver Operating Characteristic (ROC) curve analysis, performed on experimental infection samples from Figure 4. Results of this analysis is shown in Supplementary Table 2.

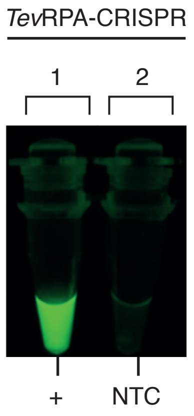

Supplementary Figure 10. Naked eye fluorescence readout of the *Tev*RPA-CRISPR test. Fluorescence was observed using a blue-light transilluminator after 120 min of reaction time.
